# Auditory verbal hallucinations as atypical inner speech monitoring, and the potential of neurostimulation as a treatment option^☆^

**DOI:** 10.1016/j.neubiorev.2013.10.001

**Published:** 2013-12

**Authors:** Peter Moseley, Charles Fernyhough, Amanda Ellison

**Affiliations:** a Psychology Department, Durham University, South Road, Durham DH1 3LE, UK; b Cognitive Neuroscience Research Unit, Wolfson Research Institute, Durham University, University Boulevard, Stockton-on-Tees TS17 6BH, UK

**Keywords:** Auditory verbal hallucinations, Inner speech, Self-monitoring, Reality monitoring, Neurostimulation, TMS, tDCS

## Abstract

Auditory verbal hallucinations (AVHs) are the experience of hearing voices in the absence of any speaker, often associated with a schizophrenia diagnosis. Prominent cognitive models of AVHs suggest they may be the result of inner speech being misattributed to an external or non-self source, due to atypical self- or reality monitoring. These arguments are supported by studies showing that people experiencing AVHs often show an externalising bias during monitoring tasks, and neuroimaging evidence which implicates superior temporal brain regions, both during AVHs and during tasks that measure verbal self-monitoring performance. Recently, efficacy of noninvasive neurostimulation techniques as a treatment option for AVHs has been tested. Meta-analyses show a moderate effect size in reduction of AVH frequency, but there has been little attempt to explain the therapeutic effect of neurostimulation in relation to existing cognitive models. This article reviews inner speech models of AVHs, and argues that a possible explanation for reduction in frequency following treatment may be modulation of activity in the brain regions involving the monitoring of inner speech.

## Highlights

• We discuss ‘inner speech’ theories of auditory verbal hallucinations.
• Atypical self-monitoring may lead to the experience of inner speech as external.
• We summarize research into the use of neurostimulation to treat hallucinations.
• Effects of neurostimulation may be due to modulation of self-monitoring networks.

## 1 Introduction

Auditory verbal hallucinations (AVHs) are the phenomenon of hearing voices in the absence of any speaker, and are experienced by around 60–80% of people diagnosed with schizophrenia (Sartorius et al., 1986). Some studies also report that they are experienced by between 1.5% and 3% of the general population (Tien, 1991), highlighting that the experience is not always pathological, though estimates greatly vary between sources on this matter (Beavan et al., 2011). Despite the prevalence of this experience, surprisingly little is known about the cognitive and neural mechanisms underlying AVHs, and they may be refractory to current treatment options in around 25% of cases (Shergill et al., 1998).

A recent review by Sommer et al. (2012) suggested that antipsychotic medication such as olanzapine, amisulpride, ziprasidone or quetiapine may be the most efficacious treatment option for AVHs in schizophrenia, while clozapine should only be used in the event that these are unsuccessful. Anti-psychotic medication tends to block D_2_-receptors in the brain, leading to hypotheses emphasizing the importance of dopamine pathways in the creation of psychotic experiences (Carlsson, 1978; Farde, 1997). However, it is well known that antipsychotic medication often causes undesirable side effects, such as weight gain and sedation (Buchanan et al., 2010). Therefore, cognitive behavioural therapy (CBT) is often used, either as an adjunctive or as an alternative treatment. The aim of CBT is to change the appraisal of the hallucination, in a collaborative effort between the patient and therapist; the patient is encouraged to take an active part in the therapy, for example, by examining evidence for and against distressing beliefs, and testing explanations for unusual experiences in real world situations (Jones et al., 2012). One meta-analysis reported an effect size of 0.4 for a reduction in positive symptoms of schizophrenia (Wykes et al., 2008), although this does not tell us specifically about CBT's efficacy in treating AVHs. These studies are also confounded by whether the patients included were taking anti-psychotic medication at the time of therapy; it is difficult to know whether any effects were due to the use of CBT alone.

The search for new treatment options for AVHs has led to the testing of the efficacy of noninvasive neurostimulation techniques in the treatment of AVHs. Although results have not been conclusive, repetitive pulse transcranial magnetic stimulation (rTMS) was recently labelled as “potentially useful” in a summary of available treatment options (Sommer et al., 2012, p. 7), and recent research has used transcranial direct current stimulation (tDCS), with promising results (Brunelin et al., 2012). Additionally, neurostimulation techniques, if indeed efficacious, have the potential to tell us much about the cognitive and neural mechanisms underlying AVHs, by targeting specific brain regions thought to be involved in the experience (although it also affects brain regions other than those directly underneath the stimulating coil or electrode; e.g. Kindler et al., 2013). There has so far been little attempt to explain the therapeutic effects of neurostimulation (if not a placebo effect) in relation to pre-existing cognitive or neuroscientific models of AVHs.

The most popular cognitive theory of AVHs is arguably that many are the result of internal cognitive events, such as inner speech, being misattributed to an external or alien source (Waters et al., 2012a). Various models have suggested that this could be due to a specific deficit in the monitoring of one's own actions, known as self-monitoring (Frith, 1992), and/or due to a bias towards labelling internal mental events as externally produced under conditions of ambiguity, known as a bias in reality monitoring (Bentall and Slade, 1985). Evidence from neuroimaging suggests that monitoring of one's own speech, overt or covert, is related to activity in auditory cortical regions such as the lateral temporal lobe, including the superior temporal gyri (STG), a brain area that includes both primary and secondary auditory cortices (Allen et al., 2007; McGuire et al., 1996a). This corresponds well to ‘symptom-capture’ studies of AVHs, in which similar areas are often implicated (Allen et al., 2008). rTMS treatment is usually targeted at the left temporoparietal junction (TPJ), an area adjacent to, and with high levels of connectivity to, primary and secondary auditory cortex (Kindler et al., 2013). Therefore, it is possible that neurostimulation treatment affects brain regions involved in verbal self- or reality monitoring.

This review will discuss models that implicate atypical monitoring of inner speech, as well as the evidence surrounding the efficacy of neurostimulation as a treatment for AVHs, and the possible cognitive and neural mechanisms behind the therapeutic effect.

## 2 Auditory verbal hallucinations as the result of misattributed inner speech

Prominent models of AVHs have suggested that the experiences arise when an internal mental event is misattributed to an external or non-self source. For example, Frith (1992) suggests that, if inner speech is not recognized as self-initiated, it may be experienced as an AVH. Many models have assumed that the raw material of AVHs is a kind of inner speech (Bentall, 2003; Fernyhough, 2004), although definitions of inner speech have varied, from simply ‘thinking in words’ (McGuire et al., 1995, p. 596) to ‘the overlapping region of thought and speech’ (Jones and Fernyhough, 2007a, p. 148), the latter of which highlights that not all thought processes necessarily take place as inner speech.

Perhaps the most compelling evidence that the raw material of AVHs is misattributed inner speech comes from studies that have used electromyography (EMG) to show subvocalization (tiny movements of the vocal musculature which occur during inner speech; Gould, 1948; Inouye and Shimizu, 1970; McGuigan, 1966) whilst patients experience AVHs. In one case, the subvocalizations were amplified into intelligible speech which corresponded well to the contents of the AVH (Green and Preston, 1981), and some AVHs have been shown to be less frequent when patients explicitly vocalized competing utterances, for example humming (Green and Kinsbourne, 1990). Further evidence from neuroimaging studies suggests that similar cortical areas are active during inner speech as during AVHs. For example, during auditory verbal imagery, Shergill et al. (2001) found activation in the left superior temporal gyrus (including Wernicke's area) and the left inferior frontal gyrus (Broca's area), as well as in the supplementary motor area (SMA) and insula. These findings concord fairly well with other inner speech functional neuroimaging studies (Friedman et al., 1998; McGuire et al., 1996b). Raij and Riekki (2012) showed that the main difference between neural activation during AVHs and during imagining speech was that AVHs showed less activation in the SMA, otherwise implying that similar areas were recruited for imagining speech and AVHs. The functional localization of inner speech has also been studied using single pulse TMS: Aziz-Zadeh et al. (2005) were able to induce ‘covert speech arrest’ by stimulating either motor or non-motor language areas in the inferior frontal gyrus (IFG) in the left hemisphere, but not right hemispheric non-motor language areas.

In contrast, however, some have argued that left hemisphere language sites are not integral to the experience of AVHs. An fMRI study using a sample of 24 hallucinating patients, concluded that the right homologue of Broca's area (IFG) and the right superior temporal gyrus, as well as the bilateral insula and anterior cingulate gyri, were most active during AVHs (Sommer et al., 2008). Vercammen et al. (2010b) have also shown that functional connectivity of the left temporoparietal junction (TPJ) with the right homologue of Broca's area is reduced in patients who reported AVHs. These findings may be interpreted as discordant with the inner speech theory of AVHs, especially in light of Aziz-Zadeh et al.’s findings, which indicate that non-motor language areas in the right hemisphere are *not* causally involved in the production of inner speech. However, there are a number of possible explanations for right hemisphere involvement in AVHs. Vercammen et al. argue that inner speech generated by the right hemisphere may consist of short sentences, with negative or derogatory content, which seems to fit with phenomenological accounts of AVHs. It may simply be that the type of inner speech elicited by Aziz-Zadeh et al. did not recruit right hemisphere language areas. Alternatively, right-sided language areas could be involved in the contextualisation of AVHs (influencing emotional valence and attentional salience, for example). This suggestion would fit with findings that implicate right hemispheric activation in emotional prosody comprehension (Alba-Ferrara et al., 2012a, 2012b). Superior temporal regions of the right hemisphere are also important in processing aspects of speech such as pitch (Lattner et al., 2005).

Alternatively, the right temporoparietal junction (rTPJ) has been implicated in theory of mind tasks (Young et al., 2010a, 2010b) and it has also been argued that the inferior parietal lobule (immediately adjacent to Wernicke's area and its right homologue), particularly on the right side, is important for feelings of self-agency (Jardri et al., 2007), leaving open the possibility that right-sided activation in AVHs is a result of the utilization of some form of perspective taking mechanism (a possibility returned to later in this section). Inconsistent neuroimaging findings in relation to the lateralization of AVHs may reflect the varying phenomenology of the experience, and it is likely that not all AVHs can be linked to inner speech (Jones, 2010).

Hoffman et al. (2011) have argued that a better model to explain AVHs involves the surfacing of ‘unbidden thoughts’ into consciousness, through a hyperconnected corticostriatal loop involving Wernicke's area and its right homologue, the left inferior frontal gyrus, and the putamen bilaterally. This model also specifies that a possible reason for the experience of AVHs as another person's voice is linked to the activation of right-sided temporal areas. Although different in its details, this model is not incompatible with the typical view of inner speech as the raw material of AVHs, additionally emphasizing the importance of subcortical structures such as the putamen in conscious experience. The putamen is crucial in the initiation of language representations (Price, 2010), and Hoffman et al. argue that hyperconnectivity of the putamen with temporal and frontal areas represents an overabundance of language representations reaching temporal cortices. It is not immediately clear, though, why these language representations might be experienced as hallucinatory and as external to the self. If anything, the differences between the unbidden thoughts model and inner speech models emphasize the need for a better understanding of the phenomenology of what we are referring to as ‘inner speech’.

It may be that the differential findings of inner speech and AVH neuroimaging studies are in fact due to the type of task used to elicit inner speech. For example, many of the aforementioned studies have simply asked participants to repeat cued sentences in their heads, whilst Aziz-Zadeh et al. (2005) inferred covert speech arrest by observing an increase in reaction time in a syllable counting task. Although these tasks undoubtedly elicit some form of inner speech, their validity in relation to the kinds of inner speech that we experience in real life, or that may be related to the experience of AVHs, may be tenuous. For example, these forms of elicited inner speech may lack spontaneity and the phenomenal experience of an inner dialogue (see below). Future inner speech neuroimaging studies would therefore do well to utilize tasks that may elicit more realistic inner speech, as discussed in Section 6.

Inner speech theories of AVHs, though, have been criticized for not explaining the phenomenological aspects of AVHs. For example, AVHs are usually experienced as non-self generated and usually (but not always) located in external space. Furthermore, most hallucinations take the form of another person's voice, often giving commands or commenting on actions of the person, and usually being experienced as ‘alien’ to the self (Nayani and David, 1996). This does not seem to correspond to what most would associate with ‘thinking in words’, and the negative and often derogatory content of AVHs would also seem to contrast with this idea. One study reported no phenomenological difference between the inner speech of hallucinating patients diagnosed with schizophrenia and a control group of participants who did not hear voices (Langdon et al., 2009), whereas one might expect to find differences in, for example, the tendency to represent others in inner speech (although, interestingly, the two questionnaire items which approached a significant effect were related to experiencing inner speech as a dialogic exchange). Also, an early neuroimaging study found no difference in brain activation between hallucinating patients and healthy controls during inner speech (McGuire et al., 1995).

Fernyhough (1996, 2004) has argued that inner speech is fundamentally dialogic in nature, or ‘shot through’ with other voices. This is a logical extension of Vygotsky's (1934/1987) argument that inner speech is the result of the internalization of external dialogues during psychological development. If true, it follows from Vygotsky's ideas that typical inner speech may consist of a dialogue, often including voices other than the person's own. One aspect of inner speech that has been shown to differ between hallucination-prone and non-hallucination prone healthy individuals is in fact self-reported propensity to use dialogic inner speech (McCarthy-Jones and Fernyhough, 2011), and the inner speech neuroimaging study by McGuire et al. did find differential activation between hallucinating patients and controls when participants were asked to imagine someone else's voice. This would seem to explain why most AVHs are experienced as a voice other than the person's own (McCarthy-Jones et al., 2012; Nayani and David, 1996): the atypical component of AVHs is not that they are experienced as someone else's voice, but instead that they are experienced as alien and/or external to the self (Jones and Fernyhough, 2007a). As already mentioned, the observation of right hemispheric activity during AVHs may in fact reflect engagement of a type of perspective-taking mechanism, which would be integral to the dialogicality of inner speech. This is backed up by the involvement of right temporal lobe involvement in theory of mind tasks (Young et al., 2010a, 2010b).

It therefore seems that the inner speech model is a good fit for at least some types of AVHs, although further research to elicit a better proxy for inner speech, and research that studies the neural correlates of different phenomenological types of AVHs (and inner speech), is needed before firm conclusions can be drawn.

## 3 Why do people who hear voices experience inner speech as alien?

If AVHs are indeed the result of inner speech being misattributed to an external/non-self source, it may follow that a mechanism that usually distinguishes between internally and externally produced stimuli is disrupted. This concept has variously been termed self-monitoring, source monitoring, or reality monitoring (Bentall, 1990; Frith, 1992). These terms have often been used interchangeably in the literature, or simply grouped under the umbrella term ‘source monitoring’. In general, self-monitoring has tended to refer to the ability to monitor the planning and executing of actions (with inner speech being seen as a motor act), and has been associated with tasks requiring participants to monitor self-made actions or vocalisations (Frith, 1992). Meanwhile, source and reality monitoring have tended to be defined as the ability to distinguish between internal and external events, and have been associated with tasks requiring participants to recall whether a remembered item was produced by themselves or the experimenter (source memory) or signal detection tasks requiring participants to decide whether a voice is present in white noise or not (Bentall and Slade, 1985; Johnson et al., 1993). Here, the terms will be used as described above, but the term ‘monitoring’ will also be used as an umbrella term to cover all of these concepts. (See Table 1 for a summary of cognitive tasks discussed in this review.)

Early self-monitoring studies measured schizophrenia patients’ ability to monitor their own actions by using a simple joystick task in which participants had to monitor errors without feedback (Frith and Done, 1989). It was shown that those diagnosed with schizophrenia were worse at this form of monitoring than healthy controls. Neuroimaging studies investigating self-monitoring of speech specifically implicated a network of brain areas, involving the lateral temporal cortex bilaterally (consistent with theories that implicate similar brain regions in monitoring both internally and externally produced speech), as well as left inferior frontal cortices and hippocampal formations (McGuire et al., 1996a). Following from early self-monitoring studies, one prominent theory has suggested that an internal forward model is disrupted in those that experience AVHs. This theory explains the feeling of agency that accompanies motor actions by postulating a system that uses the predicted consequences of actions to label events as self- or other-generated. Importantly, this theory relies on inner speech being seen as a covert motor action (Jones and Fernyhough, 2007b), which is supported by the aforementioned subvocalization research. A forward model account of self-monitoring and AVHs argues that when a motor plan is first created, an ‘efference copy’ or ‘corollary discharge’ of the plan is sent to sensory areas to ‘warn’ them that the action is about to occur (Ford and Mathalon, 2005). If the planned action then occurs (and appropriate sensory information is received as reafference), the event is labelled as self-generated (Seal et al., 2004; Wolpert et al., 1995). Some models specify that the efference copy will dampen activity in the appropriate sensory area, to label the percept as self-generated (Ford and Mathalon, 2005; Whitford et al., 2012). Applied to AVHs, this would mean that an efference copy of the inner speech motor act has not reached auditory cortical areas (i.e. Wernicke's area, and the STG more generally).

The forward model account of self-monitoring has received support from tasks that require participants to discriminate between distorted voices (lowered either 3 or 6 semitones) that could be their own or someone else's. Here, a voice is immediately fed back to them through headphones when participants speak, and they are required to respond whether they think the voice is their own or not (McGuire et al., 1996b). Studies utilizing this task have shown that patients with AVHs are worse at making the self/other judgement correctly (Johns et al., 2001). Evidence of a global self-monitoring deficit in schizophrenia also comes from studies which show that patients with AVHs do not show a difference between the tickle sensation evoked by others and by themselves, when both healthy controls and patients without AVHs do (Blakemore et al., 2000). Interpreted in light of the forward model theory of AVHs, typical individuals may not be able to tickle themselves because the corresponding sensory cortical areas are dampened when the efference copy of the motor plan successfully reaches it, whereas this may not be the case in hallucinating patients. These findings can therefore be seen to support the idea of a disrupted forward model self-monitoring system in those experiencing AVHs.

The neural instantiation of the efference copy has been postulated to be a dampened N1 event-related potential (ERP) during self-produced speech in comparison to other-produced speech in healthy controls, but not in patients with schizophrenia (Ford et al., 2001). Magnetoencephalography (MEG), the magnetic counterpart of EEG, indicates that the N1 ERP component originates in the STG (Krumbholz et al., 2003). This finding could reflect the failure of an efference copy to successfully dampen activity in sensory areas after self-produced speech, perhaps due to a delayed corollary discharge (Whitford et al., 2012). This is supported by neuroimaging evidence suggesting that left superior temporal areas are more active during inner speech in those diagnosed with schizophrenia than healthy controls (Simons et al., 2010). Further EEG studies have shown that theta and gamma band coherence between frontal and temporal areas is impaired in patients with schizophrenia, implying that synchronous neural activity may be the neural substrate of the efference copy (Ford and Mathalon, 2005). Disrupted connectivity between frontal and temporal cortical areas has often been implicated in AVHs (Lawrie et al., 2002), and is possibly linked to structural alterations in white matter tracts such as the arcuate fasciculus (de Weijer et al., 2011). Self-monitoring studies have therefore provided evidence that hallucinating patients may experience inner speech as alien because of a failure of the efference copy system to dampen activity in auditory cortex and label it as self-generated.

It has, however, been argued that a deficit in self-monitoring as measured by some of the aforementioned tasks is not enough in itself to explain the misattribution to *external* sources that has been proposed to explain AVHs, and that there must be a specific bias towards labelling events as external (Allen et al., 2004). Therefore, tasks that attempt to measure participants’ bias towards locating events externally have been used with both hallucinating and non-hallucinating patients, as well as healthy controls. These reality monitoring tasks have been used to show a so-called ‘externalizing bias’. A response bias such as this would lead to a higher likelihood of stimuli of ambiguous source being attributed to an external source (Bentall, 1990). Early tasks utilized signal detection theory, in which hallucination-prone individuals, hallucinating patients and healthy controls were asked to discriminate whether a voice was present in white noise, showed that the former two groups showed a response bias towards external misattributions (Bentall and Slade, 1985). More recent neuroimaging studies using auditory signal detection tasks have implicated, among other areas, the STG in the creation of false alarms (responding ‘yes’ when there is no voice present) (Barkus et al., 2007), therefore showing overlapping regions of activation with neuroimaging studies of both inner speech and AVHs.

A large body of research relating to reality monitoring has also accumulated looking at ‘source memory’ in people that experience AVHs. In these tasks, participants are generally required to distinguish between self-generated words, experimenter-generated words, and words that have not appeared in the task before (see Waters et al., 2012b, for a recent review of self-recognition deficits). Findings typically indicate that hallucinating patients or hallucination-prone participants are more likely to misattribute recalled items to the experimenter (Bentall et al., 1991; Laroi et al., 2004), which has again been taken as evidence that AVHs are linked to an externalizing bias. It has also been shown that patients diagnosed with schizophrenia who experience AVHs are more likely to recall an imagined word as spoken (Franck et al., 2000) or an imagined action as performed (Gawęda et al., 2013), compared to other patients and healthy controls.

Distorted voice tasks have also been used to provide evidence for the existence of an externalizing bias in those that experience AVHs. Allen et al. (2004) used a task in which, unlike the aforementioned verbal self-monitoring studies, the speech was pre-recorded. The rationale underlying this alteration was that the task would no longer measure immediate verbal self-monitoring ability, as participants were not generating the stimuli ‘online’. It was found that hallucinating patients were still more likely to make external misattributions. The authors argued that previous findings may not simply be due to a disrupted verbal self-monitoring system, but at least partly due to an externalizing bias possibly due to disrupted top-down processing of auditory stimuli. A later neuroimaging study with the same paradigm showed that, in healthy controls and non-hallucinating patients with schizophrenia, the left superior temporal gyrus was generally active when other-produced speech was listened to, whereas this was not the case when self-produced speech was listened to. These findings, however, did not apply to hallucinating patients, who did not show differential activity in this area between hearing their own or another's voice (Allen et al., 2007).

The tendency to make external misattributions may therefore be linked to additional, or alternative, mechanisms to the forward model system, because they were gained when participants were not engaged in any motor activity. Allen et al. (2007) suggest that this reflects conscious evaluation of the stimuli, perhaps involving the anterior cingulate cortex (ACC), which has strong connectivity with the temporal cortex (Petrides and Pandya, 1988). Mechelli et al. (2007) have supported this hypothesis by demonstrating a lack of effective connectivity in patients with AVHs, between STG and ACC during other-produced speech. In addition, Vercammen et al. (2010c) have demonstrated atypical functional connectivity of the ACC with left TPJ in hallucinating patients, suggesting that this connectivity may be related to a ‘core control network’ which exhibits conscious control over experiences.

Furthermore, the left planum temporale, an area within the left STG, has been shown to be involved specifically in the perception of externally located speech (Hunter et al., 2002), and posterior parts of the left STG are known to be involved in the spatial localization of speech (Mathiak et al., 2007); this area has also recently been implicated in the experience of externally as opposed to internally experienced AVHs (Looijestijn et al., 2013). Interestingly, Mathalon et al. (2001) have shown that the STG diminishes in size over time in those with a diagnosis of schizophrenia, specifically related to positive symptoms in schizophrenia, although they do not report data relating to AVHs, and so it is not possible to tell whether this finding may be specific to AVHs. It has, however, been shown that over time AVHs are more likely to be experienced as internally located (Nayani and David, 1996), although evidence is so far lacking as to whether a correlation exists between this change in STG volume and the likelihood of experiencing AVHs as internal. This evidence, though, implicates left temporal language areas as important in labelling a percept as externally located, and it follows that over-activation of this area may therefore increase the likelihood that a percept will be incorrectly labelled as external.

Temporal lobe regions, then, as well as being important in inner speech and often active during AVHs, have been implicated in both self-monitoring failures in tests of forward model theories and reality monitoring biases towards the external. This may imply that self-monitoring and reality monitoring tasks are to some extent measuring the same cognitive mechanism, although whether this is the ability to distinguish between the internal and external in space, or the ability to monitor self-generated actions and label them as self or non-self, is unknown, and is beyond the scope of this article. Returning to the discussion of neurostimulation as a treatment for AVHs: neuroimaging findings relating to AVHs, inner speech and self-/reality monitoring all point towards a key role for the left temporal lobe in the experience of AVHs (with differential findings regarding the right hemisphere). It is therefore possible that the success of the treatment may depend on its ability to modulate cortical areas involved in inner speech and self-/reality monitoring.

## 4 Neurostimulation as a treatment for AVHs

### 4.1 Neurostimulation as a therapeutic technique

Transcranial magnetic stimulation (TMS) is a noninvasive brain stimulation technique in which a coil placed on the scalp uses a rapidly changing magnetic field to induce an electrical current in the cortex (Hallett, 2007; Walsh and Cowey, 2000). Pioneered by Barker et al. (1985), TMS was at first used in single pulses, and can essentially introduce a focal area of neural noise in an area of cortex by activating neurons underlying the stimulating coil. Repetitive TMS (rTMS), in contrast, uses repeated pulses and can be applied in an event-related manner (to disrupt regions, synchronously with presented stimuli), to test whether a specific cortical area is necessary when completing a specific cognitive task (as if the area is responding to the magnetic pulses, it cannot respond to the concurrent task demands). It is worth noting that secondary areas may also be affected by the introduction of neural noise due to connectivity, and that task effects may not be related to rTMS of the primary region but functionally connected, or indeed anatomically connected, regions (Komssi et al., 2002; Walsh and Pascual-Leone, 2003). This factor is most prevalent when rTMS is utilized over longer time periods, in the absence of any task or stimuli, because it can have lasting after-effects of excitation or inhibition of cortical areas both directly underneath the coil and trans-synaptically (Hoffman and Cavus, 2002; Wassermann et al., 1998). Results showing changes in excitation or inhibition in regions distal to the stimulating coil highlight that inferences regarding the role of specific brain areas in tasks need to be made cautiously, although this may be an advantage when attempting to modulate activity in widespread cortical networks (Pascual-Leone et al., 1998).

Although TMS excites all neurons in the stimulated region with each pulse (both excitatory and inhibitory), it is important to distinguish between this and the excitation or inhibition of function that may follow. For example, low frequency (1 Hz) rTMS can have lasting after-effects, which tends to cause a decrease in neuronal activity in the stimulated region, whereas higher frequencies (>5 Hz) can cause lasting excitation (Maeda et al., 2000). The effects of TMS can also be modulated by underlying tissue type. For example, differences in anisotropy can affect the spatial distribution of the induced field, so although TMS is typically thought to largely affect grey matter, recent findings indicate that the morphology of underlying white matter tracts is also important (De Lucia et al., 2007; Opitz et al., 2013). It has further been established that TMS can have state-dependent effects, and is thought to preferentially stimulate neurons that are less active (Silvanto et al., 2008, 2007) and can also have differential effects on cortical excitability depending on baseline levels. For example, Siebner et al. (2004) showed that if excitability was increased at baseline, then 1 Hz rTMS reduced excitability; however, if excitability was decreased at baseline, the same stimulation had the effect of increasing excitability. The mechanism through which rTMS can produce lasting after-effects is still somewhat unclear, but may be due to long-term potentiation or long-term depression (LTP/LTD)-like effects, i.e. the observation that the strength of synapses between neurons can be altered if they repeatedly fire synchronously (Hoffman and Cavus, 2002). Since rTMS can have effects in regions distal to the stimulating coil, particularly when used to produce after-effects, it has the potential to affect neuronal networks thought to be involved in neurological and psychiatric conditions that may be a result of changes in connectivity between brain regions.

Typically, studies applying rTMS to test its therapeutic potential stimulate for protracted periods of time (e.g. 15 min per day, for three weeks). The intensity of stimulation is determined for each participant separately, using the individual's ‘motor threshold’ (the intensity at which stimulation of motor cortex can elicit a hand movement); for example, treatment may be administered at 90% of each individual's motor threshold. Perhaps most famously, the observation that under-activation of the left dorsolateral prefrontal cortex often coincides with clinical depression led to the use of high-frequency rTMS over this area, and there is evidence that it may be an effective treatment option (George et al., 2010, 1995). That said, some argue that the efficacy has often been exaggerated, and more studies may be needed to ensure that improvements are not simply a placebo effect (Miniussi et al., 2005; Ridding and Rothwell, 2007). The example of depression highlights difficulties in showing efficacy of rTMS as a valid treatment option, in that depression is a diverse diagnosis, and by definition is a subjective experience. This makes it hard to exclude placebo effects, especially since the control ‘sham’ condition usually used in rTMS studies has been criticized (Robertson et al., 2003). These criticisms are equally valid when applied to using rTMS to treat AVHs, and will be returned to below. Nevertheless, rTMS has now been approved for use in the treatment of depression by the Food and Drug Administration in the US (Connolly et al., 2012).

An alternative neurostimulation technique is transcranial direct current stimulation (tDCS). This can be used to selectively increase or decrease excitability of brain areas, as rTMS can. In tDCS, a weak electrical current is passed between two electrodes attached to the scalp. Current runs from the anodal electrode, under which the neurons’ membrane potentials are generally depolarized, to the cathodal electrode, under which they are generally hyperpolarized. This leads to increased neuronal excitability under the anode, and decreased excitability under the cathode (Nitsche and Paulus, 2000, 2011). Importantly, effects of tDCS which outlast the stimulation period are often observed, probably due to longer term GABAergic and glutamatergic mechanisms (Stagg and Nitsche, 2011), leading to studies into whether this technique could be used therapeutically for neurological and psychiatric disorders. One advantage of using tDCS over rTMS in an experimental setting is a more realistic sham condition. Active stimulation using tDCS leads to no more than a tingling or itching sensation underneath the electrodes, and participants tend to report that this sensation fades away after a short period of time. Therefore, sham tDCS attempts to mimic this by stimulating for only 30 s, and then gradually decreasing the stimulation intensity until the equipment is turned off. In this way, participants tend to be unaware that they are no longer receiving active stimulation (Gandiga et al., 2006; though see O’Connell et al., 2012). On top of this, tDCS is less expensive and easier to apply than rTMS, and can potentially be used by patients at their own homes, with the clinician providing indirect support with a remote trigger (Brunoni et al., 2012).

### 4.2 Can neurostimulation be used to treat AVHs?

As discussed above, neuroimaging studies using positron emission tomography (PET) and functional magnetic resonance imaging (fMRI) have shown that AVHs are often accompanied by activation of the speech and language perception areas in the left hemisphere, in agreement with inner speech theories of AVHs (Allen et al., 2012; Silbersweig et al., 1995), and research also suggests that patients with AVHs often show deficits in speech processing (Hoffman et al., 1999a). Therefore, initial studies tested the therapeutic effect of low-frequency (1 Hz) rTMS over left temporoparietal cortex (midway between the T3 and P3 electrodes using the EEG 10-20 system), at first tested on three patients diagnosed with schizophrenia (Hoffman et al., 1999b), and later on a larger sample of 50 (Hoffman et al., 2005). Hallucinating patients received rTMS treatment for 9 consecutive days (excluding weekends). These initial studies indicated that rTMS may be effective as a treatment to reduce AVHs, as measured by the Auditory Hallucinations Rating Scale, a 7-item scale which assesses hallucination frequency, number of voices, volume, vividness, salience, length and distress caused. A large effect size of .94 was found in the 50 patient sample, reducing the *frequency* of AVHs. There was no improvement in other scores relating to positive or negative symptoms of schizophrenia, implying that the effects of stimulation are relatively specific to a reduction in AVHs.

Recently, Hoffman et al. (2013) replicated their initial findings with a sample size of 83, albeit it with reduced effect size of .65 for reduction in frequency of AVHs. This effect size was increased to .74 when only patients with whom they could consistently detect a motor threshold were included. They also showed that stimulation of the right homologue of Wernicke's area could lead to a reduction in frequency of AVHs, especially for those rated high in ‘attentional salience’ (“the degree to which hallucinations capture attention and alter ongoing thoughts and behaviour”, p. 2).

Some studies, however, have failed to show the substantial improvement reported by Hoffman and colleagues. Notably, a relatively large randomized controlled trial (N = 62) failed to find any significant advantage of active rTMS over sham stimulation, despite using fMRI and image-guided stereotaxy to localize the stimulation to the point of maximal activity during each patient's AVHs (Slotema et al., 2011). Nevertheless, meta-analyses with this finding taken into account still showed positive effects of rTMS with a moderate effect size of .38 (Slotema and Daskalakis, 2012).

Whether rTMS is effective at reducing frequency of hallucinations is confounded by the fact that most studies have used either medication- or therapy-resistant patients with a diagnosis of schizophrenia. It is difficult to speculate on whether rTMS would be more or less effective if tested on drug-naive individuals, or on patients without this diagnosis, but inter-individual variability in, for example, white matter volume, could change the distribution of current induced by stimulation, as previously mentioned. Many studies also do not report the specificity of the effects of neurostimulation – that is, whether there was a corresponding reduction in other positive symptoms. This information is crucial if conclusions are to be drawn relating to the underlying mechanisms of AVHs. Nevertheless, Hoffman et al. (2005) reported that there was no significant change in the frequency of other positive or negative symptoms, and so it seems likely that neurostimulation treatment operates solely on the symptom of AVHs.

Additionally, it is possible that a publication bias has meant that negative findings with regard to efficacy are not publicly available; the effect sizes of published studies using rTMS to treat AVHs have tended to decrease with time, and so it is possible that some early negative findings were not published. Nevertheless, Slotema et al. (2012) conclude that there is little evidence of a publication bias, because there are examples in the literature of small, early studies with negative findings. Another major criticism, also made by Slotema et al., is that many studies have not achieved adequate statistical power: Hoffman et al.’s 50 patient sample, their recent 83 patient sample, and Slotema et al.’s recent negative finding being the exceptions. It is important that future studies aim to achieve higher statistical power to increase reliability.

One possible reason for the variable findings of the therapeutic effects of rTMS on AVHs is the inadequacy of the sham condition in rTMS trials. Active rTMS trials elicit a loud clicking sound, with a characteristic tapping sensation on the scalp underneath the coil. This sensation is hard to mimic realistically – although sham coils do exist, they usually do not mimic anything more than the auditory aspect of receiving rTMS. Many studies, rather than utilizing a sham coil, will tilt the active rTMS coil 45° or 90° from the scalp. In this way, both the sound and tactile sensation of rTMS are, to some extent, replicated. This method, though, leaves open questions about whether the stimulation may have some effect on underlying (or surrounding areas of) cortex. Indeed, Lisanby et al. (2001) showed that, when the coil was tilted 45° from the scalp, the voltage induced in the cortex was approximately 33% of that induced in the ‘active TMS’ condition. It is also unclear to what extent this technique is successful in blinding participants to the condition they are in: Hoffman et al. (2005), using this sham technique, reported that many patients correctly guessed which condition they had been in, but argued that in most cases their guess was actually based on curtailment of symptoms. Nevertheless, this is anecdotal, and the fact that many patients were able to tell which condition they were in may have affected the results.

It has therefore been suggested that in experiments showing a positive effect, patients receiving rTMS treatment in fact showed a placebo effect to the treatment, with the observed difference between conditions being due to an inadequate sham condition (Slotema et al., 2011). Further, the essentially subjective measures of severity of hallucinations arguably leave the studies even more susceptible to being confounded by the placebo effect. Some measures, however, are less subjective than others; Hoffman et al. (2013) asked participants to record frequency of AVHs with a mechanical counter, perhaps negating this criticism. In addition, most rTMS studies have recruited patients whose AVHs have been refractory to anti-psychotic drugs or other treatment options, leaving open the question of why the placebo effect would be evident after rTMS, but not other attempts to reduce AVHs.

No studies to date have looked at the efficacy of rTMS in the treatment of different types of AVH, but there is a growing realization that AVHs cannot be treated as one homogeneous group, and may in fact differ both phenomenologically and in their cognitive and neural substrates (Jones, 2010; Nayani and David, 1996). Recent studies, for example, have suggested a subtype of AVH known as ‘hypervigilance hallucinations’, characterized by their occurrence when attention is externally focused (Garwood et al., 2013). That phenomenologically different AVHs may have different neural substrates is highlighted by Hoffman et al.’s most recent study (2013), showing that the effectiveness of rTMS to the right hemisphere is dependent on attentional salience of the AVHs. It is therefore possible that only some AVHs may be amenable to treatment using rTMS. If this were the case, the results may be skewed depending on the ‘types’ of AVHs that were studied. It will be argued in Section 4.3 that AVHs which may be best described as misattributed inner speech may be most amenable to neurostimulation treatment.

At the time of writing, only one experimental study of the therapeutic effect of tDCS on AVHs has been reported. Brunelin et al. (2012) studied 30 hallucinating individuals diagnosed with schizophrenia, placing the cathodal electrode over the left temporoparietal junction (midway between the T3-P3 electrodes as specified by the 10-20 EEG system, similarly to rTMS studies), and the anodal electrode over left dorsolateral prefrontal cortex; abnormal white matter volume of this area is often associated with negative symptoms of schizophrenia (Sanfilipo et al., 2000). The participants underwent stimulation twice a day, for 20 min, for 5 days. Half of the participants were assigned to the sham condition, while half were assigned to receive active stimulation. Results showed that those who received active cathodal stimulation over temporoparietal cortex experienced a 31% reduction in hallucination severity, as measured by the Auditory Hallucination Rating Scale (which takes into account variables such as hallucination frequency, loudness, and salience of the AVH), compared to an 8% reported reduction in the sham condition. This effect was still present 3 months later, with 6 of 15 participants in the experimental condition showing a reduction in hallucination frequency of more than 50%. The most obvious criticism of this study is the relatively small sample size; it will be interesting to see whether future studies are able to replicate these results. As already mentioned, the sham condition used in studies using tDCS is more effective, and therefore may be less susceptible to placebo effects. Issues such as portability and ease of use may also make it more realistic as a treatment option.

To summarize, the evidence regarding efficacy of noninvasive brain stimulation techniques as a treatment for AVHs is still equivocal. What follows is an analysis of how treatment of AVHs with neurostimulation may affect the associated cognitive and neural mechanisms, interpreted within an inner speech monitoring framework.

### 4.3 How might treatment with neurostimulation affect the cognitive and neural mechanisms associated with AVHs?

Previous attempts to treat AVHs with noninvasive brain stimulation have not been carried out based on a clear prediction from our understanding of the cognitive mechanisms underlying AVHs. Recent evidence, however, suggests that neurostimulation may be effective as a treatment option due to its effects on brain networks involved in the monitoring of inner speech. The following section aims to interpret the findings discussed in Section 4, based on inner speech models of AVHs, and discuss recent studies which have used neuroimaging to monitor the after-effects of neurostimulation in cortical regions known to be involved in AVHs. Table 2 provides a summary of some key findings regarding the importance of different brain areas in AVHs and neurostimulation treatment.

One of the few studies to look at the effects of left temporoparietal rTMS on reality monitoring performance in those that experience AVHs was conducted by Brunelin et al. (2006). This study used an rTMS protocol similar to those used in other studies of rTMS to treat AVHs, and was able to replicate the improvements in Auditory Hallucination Rating Scale score shown by others. The 24 patients also took part in a source memory test in which they had to recall whether they had read an item silently to themselves, or said the word out loud (a ‘say/imagine’ paradigm). Patients that received active rTMS were less likely to misattribute an imagined word as one they had said after stimulation, whereas those that were allocated to the sham condition did not show this pattern. This can therefore be seen to support both the efficacy of rTMS as a treatment option for AVHs, and the link between AVHs and reality monitoring.

The importance of the STG in responders to neurostimulation treatment has also been highlighted by a recent neuroimaging study showing higher left STG activation in a pre-TMS resting-state scan in those that were later classified as ‘responders’ to the TMS treatment paradigm (Homan et al., 2012). These findings may be crucial, as they suggest that pre-existing levels of activity in the STG may be one biomarker for recognizing likely responders to neurostimulation treatment, and are also consistent with findings discussed in Section 4.1 showing that the after-effects of rTMS are dependent on baseline levels of excitability. In a separate study, measurement of cerebral blood flow post-TMS treatment, relative to pre-treatment, showed reductions in activation in primary auditory cortex (part of the STG), Broca's area and the cingulate gyrus after 10 days of rTMS treatment (Kindler et al., 2013). This provides support for the claim that left temporoparietal cortex (the site of stimulation) is the most appropriate location to affect other temporal regions, as well as cingulate areas that may be related to conscious evaluation of stimuli. Kindler et al. suggest that a high level of activity in the STG makes it harder to differentiate between inner speech and externally perceived speech, concordant with self- or reality monitoring cognitive models of AVHs. This evidence is also consistent, to some extent, with some forward model theories of AVHs which argue that a failure of the efference copy mechanism leads to an over-active auditory cortex, therefore leading to self-produced speech being labelled as ‘non-self’ (although it neither supports nor contradicts ideas surrounding the cause of over-activity). Importantly, only reduced activation in the STG correlated with a reduction in AVHs, implying that effects in other areas may be not be causal to the improvement. The authors argue that the activation of Broca's area typically seen during AVHs is due to the production of (subsequently misattributed) inner speech, although whether the observed reduction in activity post-TMS treatment corresponds to any change in the experience of inner speech (phenomenological or quantitative) is unknown.

An excellent addition to these studies, in our opinion, would have been to investigate if any phenomenological differences in AVHs existed between the responders and non-responders to rTMS treatment. For example, it is possible that only AVHs that are best categorized as ‘misattributed inner speech’ may be amenable to this form of treatment. This hypothesis is consistent with imaging studies of self- and reality monitoring studies that show that activation of this area is related to task performance, as well as activation seen when participants are engaged in inner speech, as outlined in Sections 2 and 3. Hoffman et al.’s finding (2013) of efficacy of right-sided stimulation in more salient AVHs further implies that phenomenologically different AVHs may need to be accounted for by different cognitive-neuroscientific models. Alternatively, it is possible that a higher level of activation in right-sided temporal areas leads to a higher level of attentional salience because of other phenomenological variables, such as emotional valence.

Results from rTMS treatment protocols, however, have also been used to argue that cortical areas involved in self-agency (as measured by self-monitoring and reality monitoring tasks) can in fact be dissociated from those involved in AVHs. Jardri et al. (2009) showed that low-frequency stimulation of the right TPJ could improve performance on both types of task, but not decrease frequency of AVHs; meanwhile, left TPJ stimulation achieved both. The data, though, was gained from only a single participant (a child diagnosed with childhood-onset schizophrenia), and so further research is needed to support this finding. Despite this, the authors suggest that self-agency may be linked to a network dissociable to that drawn upon during AVHs, implicating the inferior parietal lobule (IPL) rather than the TPJ/STG per se. Previous studies have suggested bilateral involvement of the IPL in feelings of agency, with a right sided dominance (Farrer et al., 2003; Jardri et al., 2007). The IPL is immediately adjacent to the posterior STG (including the planum temporale and Wernicke's area), and may be part of an alternative pathway that runs laterally to the arcuate fasciculus between Wernicke's and Broca's area (Catani et al., 2005; Frey et al., 2008). It is possible that the IPL is part of a feed-forward mechanism between speech production areas and temporal areas (Rauschecker and Scott, 2009), which may explain evidence of its involvement in feelings of agency and monitoring tasks. It is therefore difficult to ascertain whether concurrent improvement in monitoring tasks and AVH frequency is due to the effect of stimulation on the targeted area (left TPJ) and its connections with other auditory cortical areas, or the immediately adjacent IPL, due to either the limitations of the spatial resolution of rTMS (approximately 1 cm^3^, dependent on coil size), or connectivity between the STG and the left IPL.

This is difficult to reconcile with imaging studies of monitoring tasks and symptom-capture studies of AVHs, in which superior temporal regions, particularly on the left, are often shown to be important. Although inferior parietal regions have been reported as involved in the occurrence of AVHs (Jardri et al., 2011; Lennox et al., 2000), one might expect more reliable activation of this area in neuroimaging studies of AVHs if it were of such key importance. Speculations regarding the importance of the IPL in monitoring and AVHs therefore need to be empirically tested with larger sample sizes and a variety of neuroscientific and cognitive tests, before firm conclusions can be drawn.

### 4.4 Neurostimulation and inner speech

It is interesting that Hoffman et al. (2007) found no reduction in AVHs after stimulating Broca's area, but did show that responders to the rTMS treatment tended to show reduced functional connectivity between left TPJ and the right homologue of Broca's area, supporting Vercammen et al. (2010a), who found reduced connectivity between these areas in patients with AVHs. In combination with Aziz-Zadeh et al.’s (2005) results showing induction of covert speech arrest during Broca's area stimulation, the lack of improvement shown after treatment through Broca's area stimulation may support arguments that there is no difference, at a neural level, in the *production* of inner speech in those that experience AVHs. Instead, it is the subsequent perception (by temporal regions) or evaluation (by ACC) that leads to a misattribution. In combination with the aforementioned study by Homan et al. (2012), these results indicate that reduction of activity in superior temporal regions is crucial to the therapeutic effect of rTMS on AVHs. More detailed studies are required to distinguish whether reduction in activity in specific areas of the STG, such as the planum temporale, are responsible for the improvement in AVH frequency.

The role of right hemispheric language areas in inner speech and AVHs is still a relatively unexplored area: current inner speech theories might predict that they are not integral to the creation of AVHs, but are instead important in their contextualisation (experiencing AVHs as another person's voice and/or perspective, emotional content of AVHs). Nevertheless, neurostimulation treatment protocols reviewed above suggest that in some cases stimulation of the right TPJ may be successful in reduction of AVH *frequency*, which may imply a causal role for right temporal regions in the experience of AVHs, rather than simply contextualisation. Further research into the neural basis of different forms of inner speech (e.g. dialogic, emotional) could help to clarify what role the right homologue of Broca's area and right superior temporal regions play in the generation of inner speech, self-/source monitoring, and therefore AVHs.

## 5 Future directions

The evidence so far reviewed suggests that the efficacy of neurostimulation as a treatment option may depend on its ability to modulate activity in superior temporal cortical regions, as well as inferior frontal and anterior cingulate regions. We have argued that this is consistent with inner speech theories of AVHs, which postulate that atypical monitoring processes lead to its misattribution to an external or non-self source. This is supported by findings implicating similar regions in the monitoring of speech as those affected by neurostimulation of TPJ. There are, however, a number of key avenues of research which remain to be explored.

While it seems likely that, at a cognitive level, the effect of treatment may be due to an improvement in self- and/or reality monitoring, it is hard to discount the possibility that a decrease in AVH frequency may lead to improvements in monitoring capability, perhaps due to the distracting effects of AVHs. Future studies should aim to test this more directly. Such studies could use low frequency rTMS, or tDCS, in conjunction with previously used monitoring tasks in both clinical and non-clinical populations, to study the effects of reduction of STG activity on the ability to distinguish between internal and external events. Such studies would not only be able to test the importance of superior temporal regions in monitoring tasks, but would also be able to inform us of the causality of observed improvements. They would also potentially provide evidence both for cognitive models that specify monitoring deficits or biases as a cause of AVHs, and clinical studies of the efficacy of brain stimulation techniques that claim to be able to reduce AVH frequency.

As already noted, it is likely that AVHs are not a homogeneous phenomenon (Jones, 2010). The experience may differ, for example, in terms of level of externality (the extent to which the voice is experienced as coming from the external environment), the number of voices heard, the volume of the voice, and attentional salience (the extent to which the voice captures the attention of the person, and is thus effective in altering behaviour). As yet, only a small number of studies have investigated differences in neural activity between phenomenologically different AVHs using fMRI (Looijestijn et al., 2013; Vercammen et al., 2010a). Hoffman et al. (2013) has noted that attentional salience appears to be one marker for likely response to neurostimulation treatment to the right TPJ (as opposed to the left TPJ).

On a broader level, it is possible that some AVHs can best be described as misattributed inner speech, whereas others might best be described as intrusive memories (Waters et al., 2006), and others still as ‘hypervigilance’ towards detecting stimuli in the environment (Dodgson and Gordon, 2009; Garwood et al., 2013). This review has focused on the former; that is, AVHs which seem to be explicable within an inner speech framework. As outlined, it may be that neurostimulation of the TPJ affects mechanisms that are involved with the monitoring of inner speech. An important area for future research would therefore be to investigate whether these subtypes of hallucinations are distinguishable by neural activation, and whether some types of AVHs are more amenable to treatment with neurostimulation.

Thirdly, there is a need to develop more valid inner speech paradigms if we are to understand its relation to AVHs. For example, ideas surrounding the dialogic nature of inner speech are yet to be tested within a cognitive-neuroscientific framework – important questions to address would be related to differences in activation between monologic inner speech (inner speech that does not involve the back-and-forth of a conversation) and dialogic inner speech. Moreover, it is important that more *realistic* forms of inner speech are studied. Currently, most studies rely on asking participants to repeat sentences to themselves (e.g. McGuire et al., 1995) or requiring participants to count syllables (e.g. Aziz-Zadeh et al., 2005). More valid forms of inner speech could be evoked by, for example, asking participants to imagine a conversation, evaluate behaviour, or plan a speech for a future event – these have all been suggested as important functions of inner speech (McCarthy-Jones and Fernyhough, 2011), and so should be more accurate approximations. A further possibility would be to use experience sampling techniques such as Descriptive Experience Sampling, which involves fitting participants with a ‘beeper’ which randomly cues the participant to report their current inner experience (Hurlburt and Heavey, 2001). Coupled with neuroimaging techniques, this could become a powerful method by which to investigate the neural mechanisms underlying inner speech and AVHs.

Importantly, there is a need for larger scale tests of treating AVHs with neurostimulation. Of the three studies discussed here that have achieved adequate power, one finds no effect of neurostimulation (Slotema et al., 2011), and the other two originate from the same institution (Hoffman et al., 2005, 2013). Further replication studies by independent teams are needed, and tests should be consistent with the methods used to target the stimulation (e.g. consistently using structural or functional MRI scans to locate TPJ, or following the EEG 10-20 system). It is important that studies attempt to monitor possible effects of neurostimulation of left TPJ on a wide variety of variables. Currently, evidence suggests that there are no negative effects on neuropsychological measures such a short-term verbal memory (Hoffman et al., 2005) or measures of hearing function such as pure-tone audiometry (Schonfeldt-Lecuona et al., 2012), but it would also be interesting to study potential changes in phenomenal characteristics of inner speech following reduction in activity in either left or right superior temporal regions. Finally, as outlined, testing the efficacy of tDCS to treat AVHs is a promising area of research and studies with larger samples are needed to examine whether this technique could be a useful addition to currently available treatment options for those who seek help in relieving the distress of AVHs.

## 6 Conclusions

Using neurostimulation as a treatment option for AVHs seems promising. Existing findings indicate that over-activation of the STG in the resting state is one marker for a response to the treatment. If it is possible, finding phenomenological markers of likely responders would not only mean that treatment could be targeted quickly and easily to those who might benefit most, but would also tell us much about the underlying cognitive neuroscience of AVHs. Although controversy still exists as to whether the putative therapeutic effects of rTMS can simply be attributed to an ineffective sham condition, future studies, especially those using tDCS, could settle this debate. Indeed, if noninvasive brain stimulation techniques are to be taken seriously as a viable treatment option, tDCS is a much more realistic alternative, due to the portability, ease and comfort of use, and cost. Further study is also needed into the long-term effects on AVHs of this treatment – currently, minimal evidence exists into the effects past one month.

Models of AVHs that suggest self-monitoring deficits or reality monitoring biases, leading to the misattribution of inner speech to an external or non-self source, do seem to be supported by studies using brain stimulation techniques. Although it is important not to overstate the power of neurostimulation as an experimental technique, neuroimaging studies of both hallucinating individuals and of individuals performing monitoring tasks point to the importance of left superior temporal regions and areas connected functionally and anatomically to it, in these processes. Typical neurostimulation protocols, meanwhile, direct the stimulation to affect these areas. There is tentative evidence that improvement in AVH frequency following rTMS coincides with improvement on monitoring tasks, although much more work needs to be carried out in this area to establish a causal link between the two.

## Figures and Tables

**Table 1:** Summary of cognitive tasks associated with self-monitoring and reality monitoring, and their association to AVHs.

Task	Description	Key findings	Key references
Error monitoring	Participants are asked to monitor their own actions whilst moving a joystick. The proportion of errors corrected is the variable of interest, on the basis that an internal monitor is needed to correct errors made without feedback.	Patients diagnosed with schizophrenia correct errors less often. (Not specific to AVHs.)	Frith and Done (1989)
Distorted voice	Participants listen to recordings of their own voice, and another person's voice. These recordings are sometimes distorted in pitch, and participants respond as to whether they think the voice belongs to them, or not.	Patients with AVHs are more likely to incorrectly respond that a voice belongs to someone else. This finding holds whether the voice is instantly fed back whilst the participant talks, or if it is played back at a later point in time.	Allen et al. (2004) Johns et al. (2001) McGuire et al. (1996a)
Self-experimenter word production (memory)	Participants must recall whether a word was said by themselves or the experimenter. This task is ‘offline’, in that it tests performance through *memory* of how an action was performed.	Patients with AVHs are more likely to incorrectly attribute words as produced by the experimenter.	Bentall et al. (1991) Laroi et al. (2004)
Say-imagine word production (memory)	Participants must recall whether they said a word out loud, or imagined it. Alternatively, they may be asked to perform an action, or imagine performing it. This task is ‘offline’, in that it tests performance through *memory* of how an action was performed.	Patients with AVHs are more likely to incorrectly recall saying a word out loud, or recall performing an action, as opposed to imagining it.	Franck et al. (2000) Gawęda et al. (2013)
White noise signal detection (SDT)	Participants listen to bursts of white noise, and must respond, using a button press, whether they think a voice is present in the noise.	Patients with AVHs, and hallucination-prone individuals, make more ‘false alarm’ responses (hearing voices in white noise that are not present). This seems to be due to a response bias, as opposed to a change in perceptual sensitivity.	Barkus et al., 2007, 2011 Bentall and Slade (1985)

**Table 2:** Summary of brain areas that are important in understanding the effects of neurostimulation on AVHs, and their connectivity with other regions.

Brain region	Role in AVHs	Relevance to neurostimulation treatment	Connectivity
Superior temporal gyrus (STG)	Includes PAC, Wernicke's area, and planum temporale. Structural abnormalities and functional activity consistently implicated in AVHs, and during monitoring tasks.	Posterior STG activity reduced after neurostimulation; this correlates with reduction in AVH severity.	Strong connectivity with TPJ, and effective connectivity with ACC. Also connected to IFG through arcuate fasciculus white matter tract.
Inferior frontal gyrus (IFG, Broca's area)	Crucial for production of speech (including inner speech), particularly in the left hemisphere. Role of right IFG still relatively unexplored.	rTMS of Broca's area does not lead to a reduction in AVH frequency. Reduction in activity in IFG following stimulation of left TPJ, though not correlated with reduction in AVH frequency.	Connected to STG through arcuate fasciculus white matter tract. Excessive functional connectivity with putamen in voice-hearers.
Anterior cingulate cortex (ACC)	Activation seen during AVHs may reflect conscious evaluation of stimuli, and in combination with STG, may be involved in monitoring processes.	Reduction in activity in ACC following stimulation of left TPJ, though not correlated with reduction in AVH frequency.	Connectivity with STG and TPJ may reflect verbal monitoring processes – effective connectivity during monitoring task is reduced in voice-hearers.
Inferior parietal lobe (IPL)	Often activated in symptom-capture studies of AVHs, and commonly linked to feelings of self-agency.	Data from neuroimaging has not implicated changes in activation post-neurostimulation; however, close proximity could mean activity is modulated by TPJ stimulation.	May be part of an alternative pathway that runs laterally to the arcuate fasciculus, between IFG and STG.
Putamen	Hoffman's corticostriatal loop model specifies that an overabundance of language representations initiated by the putamen may surface ‘unbidden thoughts’ as AVHs, due to hyperconnectivity with STG and IFG.	If Hoffman's model is supported, disruption of hyperconnectivity with this region may be related to the therapeutic effect of neurostimulation.	Excessive functional connectivity with IFG and STG in voice-hearers.

STG, superior temporal gyrus; PAC, primary auditory cortex; TPJ, temporoparietal junction; ACC, anterior cingulate cortex; IFG, inferior frontal gyrus; IPL, inferior parietal lobe.
